# Dietary Behaviors and Beliefs in Patients with Inflammatory Bowel Disease

**DOI:** 10.3390/jcm12103455

**Published:** 2023-05-14

**Authors:** Małgorzata Godala, Ewelina Gaszyńska, Łukasz Durko, Ewa Małecka-Wojciesko

**Affiliations:** 1 Department of Nutrition and Epidemiology, Medical University of Lodz, 90-752 Lodz, Poland; 2 Department of Digestive Tract Diseases, Medical University of Lodz, 90-153 Lodz, Poland

**Keywords:** dietary behaviors, dietary beliefs, IBD, Crohn’s disease, ulcerative colitis, diet

## Abstract

Introduction: Due to a lack of clear dietary guidelines, patients with inflammatory bowel disease (IBD) self-impose dietary restrictions based on their own nutritional experiences. The aim of this study was to investigate dietary perceptions and behavior in IBD patients. Materials and methods: A total of 82 patients (48 with Crohn’s disease and 34 with ulcerative colitis) participated in this prospective, questionnaire-based study. Based on a literature review, the questionnaire was developed to investigate dietary beliefs, behaviors and food exclusions during IBD relapses and remission. Results: The majority of patients (85.4%) believed that diet can be a trigger factor for IBD relapses, and 32.9% believed that diet initiates the disease. The majority of patients (81.7%) believed that they should eliminate some products from their diets. The most often-pointed-out products were spicy and fatty foods, raw fruits and vegetables, alcohol, leguminous foods, cruciferous vegetables, dairy products and milk. Most patients (75%) modified their diets after diagnosis, and 81.7% imposed food restrictions to prevent IBD relapses. Conclusions: The majority of patients avoided certain foods during relapses as well as to maintain remission of IBD, basing this on their own beliefs, inconsistently with current scientific knowledge. Patient education should be a key determinant in IBD control.

## 1. Introduction

Crohn’s disease (CD) and ulcerative colitis (UC) belong to a group of chronic inflammatory bowel diseases (IBDs) of unknown etiology and with increased risk of colorectal cancer. Due to a heterogeneous pathogenesis, they have diverse clinical courses, which causes diagnostic difficulties despite the availability of many multidisciplinary tests. To establish a final diagnosis, it is necessary to perform endoscopic and histopathological examinations; however, laboratory tests, such as C-reactive protein and stool calprotectin, that reflect the extent and severity of the inflammatory process are useful in the initial diagnosis [1,2,3]. Computed tomography, magnetic resonance imaging and positron-emission tomography may also be helpful. Nevertheless, endoscopy with histopathological assessment of collected specimens is essential for diagnosis [4,5,6,7].

In the last fifty years, the incidence of inflammatory bowel disease has increased significantly. IBD is a serious problem in highly developed countries, including the United States, Canada and those in Western Europe [2,8,9,10]. The latest data showed that in Poland, there are 23,574 patients with CD and 73,235 with UC. The prevalences of CD and UC are 61.6 and 191.4 per 100,000, respectively. They are reported to be higher in men than in women. The incidence of CD is 4.7 per 100,000, and that of UC, 12.5. These diseases mainly affect young people [10,11], and in the last fifteen years, a significant increase in morbidity has been observed among adolescents aged 14–17 years [12,13].

Despite recent advances in diagnosis and treatment of IBD, malnutrition remains a serious clinical problem in this group of patients. Nutritional status disorders affect about 20–70% of patients, requiring implementation of supplementary nutritional management [3,5,7,14]. There is no unified nutritional protocol for patients with IBD, and dietary management depends on the patient’s preference and is adapted to the period of the disease. Apart from pharmacological and surgical treatment, implementation of appropriate nutritional therapy is one of the most important elements in management of the disease, as indicated, among others, in the guidelines of ESPEN (European Society for Clinical Nutrition and Metabolism) and ECCO (European Crohn’s and Colitis Organisation) [15,16,17]. Patients with IBD often suffer from reduced appetite, diarrhea, impaired absorption of nutrients and resultant malnutrition. The disease causes body weight loss and failure to thrive in children and adolescents [18,19,20,21,22,23,24,25,26].

Due to the lack of clear, uniform guidelines that would apply to all individuals suffering from IBD, as well as different tolerances of food products and nutrients, patients recognize food intolerances by trial and error, based on their own nutritional experiences. Studies have shown that 75% of patients self-impose dietary restrictions based on subjective intolerance and disease severity [27,28,29]. This increases the risk of nutrient deficiencies caused by rigorous elimination diets that do not guarantee clinical improvement. Self-control of inflammatory bowel diseases should be supported with easy access to professional nutritional guidelines, and knowledge of patients’ beliefs and attitudes about nutrition may help in developing appropriate nutritional recommendations.

The aim of this study was to investigate dietary perceptions and behaviors in patients with IBD compared to scientific data and recommendations of evidence-based guidance.

## 2. Material and Methods

### 2.1. Materials

This prospective, questionnaire-based study included a total of 82 adult patients recruited from the Department of Digestive Tract Diseases, Medical University of Lodz. The diagnosis of IBD was based on each patient’s history, supported by clinical documentation. All of the subjects had been diagnosed with IBD, which was confirmed with endoscopy and histological examinations.

### 2.2. Methods

Based on a literature review concerning dietary habits of IBD patients, a questionnaire was developed [30,31,32,33,34]. It contained questions relating to patient demographics and disease characteristics (six questions), dietary beliefs (six questions), dietary behaviors (sixteen questions) and food exclusions during IBD relapses and remission (nine food groups). The questionnaire interviews were collected by a trained interviewer between June and December of 2022. The response rate for the questionnaire was 100%. This study was approved by the Bioethics Committee of the Medical University of Lodz.

### 2.3. Statistical Analysis

A statistical analysis was performed using the Statistica v.13 program. Descriptive statistics were carried out, using mean and standard deviations for continuous variables and frequency and percentages for nominal variables. The authors conducted an analysis of compatibility of the normal distribution of variables using the Shapiro–Wilk test. The means of two independent groups were compared using the chi-square test with the Yates correction, and *p* < 0.05 was adopted as significant.

## 3. Results

### 3.1. General Characteristics of the Study Participants

General characteristics of the study participants are summarized in Table 1. Of the 82 patients, 40 (48.8%) were men, the mean age was 38.1 ± 11.6 years, 48 (58.5%) had Crohn’s disease, 34 (41.5%) had ulcerative colitis and the mean duration of disease was 8.4 ± 5.7 years. High levels of education were reported by 39 (48.1%) patients, secondary levels by 31 (38.3%) and primary levels by 11 (13.6%). Fourteen (17.1%) patients were current smokers.

### 3.2. Patients’ Beliefs

We asked the respondents whether, in their opinion, food was a risk factor for IBD. Twenty-seven (32.9%) respondents believed that diet initiated the disease, and 70 (85.4%) respondents believed that diet could be a trigger factor for IBD relapses. Dietary habits were recognized as more important than pharmacotherapy in disease control by 12 (14.6%) patients (Table 2).

Fifty-four (65.9%) patients declared that in their opinion, their diets may have led to some nutritional deficiencies, and sixty-seven (81.7%) of them believed that they should eliminate some food products. The most often-mentioned products were spicy foods (65.7%), fatty foods (49.3%), raw fruits (35.8%) and vegetables (28.4%), alcohol (47.8%), legumes (59.7%), cruciferous vegetables (47.8%), dairy products and milk (47.8%).

### 3.3. Patients’ Behaviors

We analyzed the patients’ dietary habits. Forty-four (53.7%) respondents declared that they followed specific diets. Fifteen (28.9%) patients were on lactose-free diets, whereas twenty-five (65.8%) were on lactose-free diets but included cheese and yogurt. All of them imposed dietary restrictions after diagnosis of IBD. Two patients (5.3%) had celiac disease and were on gluten-free diets (Table 3).

Thirty-seven (45.1%) patients were taking vitamin and mineral supplements. Vitamin D, Ca and Zn supplements were the most often-mentioned. Only 15 (18.3%) patients were using probiotics; none of the patients was receiving any alternative therapies or herbal medications for IBD.

We asked whether the patients had modified their dietary habits after IBD diagnosis. Sixty-two (75.6%) respondents declared that they had modified their diets. The majority of patients (n = 67, 81.7%) admitted that they had imposed food restrictions to prevent IBD relapses. Among the group of 55 subjects, 35 (52.2%) individuals always adopted this strategy, whereas 20 implemented it (29.9%) often.

Reduced pleasure in eating and decreased appetite were reported by 62 (75.6%) respondents. We asked the patients to evaluate their appetite in relapse and remission of IBD, applying a scale of 0 to 10, where 10 indicated a good appetite. The mean appetite scores during relapse were 3.3 ± 2.6, whereas during remission, they were 8.7 ± 1.7 (*p* < 0.05).

Most patients (n = 62, 75.6%) declared that certain foods exacerbated their symptoms, such as abdominal pain, diarrhea, gas and bloating, during IBD relapse. Only 12 patients (14.6%) declared that some foods alleviated their symptoms. The majority of the studied patients shared meals with other members of their families (n = 76, 92.7%). The minority (n = 20, 24.4%) refused eating outside for fear of suffering relapses/relapse occurrences. Most patients (n = 54, 65.9%) declared skipping one whole meal daily during an IBD relapse, whereas only nine (11.0%) patients declared doing this during remission.

As far as dietary recommendations are concerned, 25 (30.5%) patients declared having received nutritional advice. The most popular sources of information were dietitians (n = 14, 56%), general practitioners or gastroenterologists (n = 10, 40%); family members; or other IBD patients (n = 1, 4%). The majority of patients (n = 55, 67.1%) sought information about IBD diets on their own. The most often-indicated sources of reference were the Internet (n = 51, 92.7%), books and professional articles (n = 4, 7.3%). We asked the patients to evaluate those recommendations using a scale from 0 to 5. Nutritional advice received from specialists, family members and other patients were assessed as 3.7 ± 1.2, whereas information obtained from the Internet, books and professional articles were assessed as 3.3 ± 0.9 (*p* > 0.05).

### 3.4. Food Exclusions

The majority of patients eliminated different kinds of food both during IBD relapses (n = 74, 90.2%) and during remission (n = 72, 87.8%). The foods most often eliminated during IBD relapses were spicy foods (n = 50, 67.6%) and fatty foods (n = 49, 66.2%), as well as milk and dairy products (n = 47, 63.5%). Those most often eliminated during IBD remission were spicy foods (n = 41, 56.9%), milk and dairy products (n = 39, 54.2%) and fatty foods (n = 38, 52.8%) (Table 4).

### 3.5. Dietary Habits According to IBD Subtype, Gender, Level of Education and Age

As compared to the UC group of patients, more CD patients were on lactose-free diets (27.1% vs. 5.9%, *p* < 0.05) and fewer were taking vitamin and mineral supplements (35.4% vs. 58.8%, *p* < 0.05). A significantly larger proportion of the CD patients believed that improper diet had a role in initiating the disease (37.5% vs. 23.5%, *p* < 0.05), and even more of them thought some foods should be avoided to prevent disease relapses (91.7% vs. 67.6%, *p* < 0.05).

More female respondents, compared to male respondents, had modified their diets after diagnosis of IBD (90.5% vs. 60%, *p* < 0.05) and believed that their diets could lead to nutritional deficiencies (88.1% vs. 42.5%, *p* < 0.05).

More patients with higher education, as compared to those with primary and secondary levels of education, were of the opinion that their diets could cause nutritional deficiencies (42.5% vs. 23.8%, *p* < 0.05), and fewer subjects believed that diet could cause the disease (25% vs. 40.5%, *p* < 0.05).

Age was not found to be a significant predictor of dietary behaviors.

## 4. Discussion

According to our best knowledge, this is the first Polish study to investigate dietary behaviors and beliefs in adult patients with IBD. These results demonstrate that IBD patients perceive food as a factor influencing the natural course of the disease, although scientific evidence is still lacking.

Our study demonstrates that 32.9% of patients believe that diet can initiate IBD. In contrast to our results, a French study by Zallot et al. showed that only 16% of IBD patients shared this opinion [34]. Similar observations were made in a study by Murtagh et al., in which 20% of patients with IBD indicated diet as a risk factor for the disease [35]. These findings are not supported by the study by Limdi et al., where 48% of patients with IBD perceived diet as an initiating factor in IBD [33]. Similar results were reported in a study by Crooks et al., in which 51% of the participants believed that diet was the initiating factor for their IBD [32]. In a study by Tomar et al., 44% of the subjects perceived food as a risk factor for IBD [36].

Although one-third of our respondents confirmed that diet could initiate the disease, the majority (85.4%) claimed that it could be a trigger factor for IBD flares. Similar results were found in other studies [32,33,34]. The majority of our patients declared that they had imposed food restrictions to prevent IBD relapses. Additionally, 81.7% patients believed that some products should be eliminated to prevent disease relapses, although such an approach was not recommended by specialists. Similar results were found in a study by Larussa et al., in which 70% of patients were on self-prescribed elimination diets [29]. In the study by Limdi et al., 66% of the participants gave up on their favorite foods in order to prevent relapses [33]. In the study by Murtagh et al., 85% of patients with IBD imposed dietary restrictions on themselves, hoping that it could prevent them from suffering flares [35].

The potential role of diet in initiating IBD or causing relapses needs further investigation in large prospective studies. As far as our study is based on beliefs and perceptions, it cannot be linked directly to disease behavior. There are no scientific data to prove that IBD can be initiated by diet alone. Neither is there any “IBD diet” that can be generally recommended to promote remission in IBD patients with active disease [5]. Patients probably resign from eating different kinds of food as results of their own experiences, mainly from periods of relapses, continuing this elimination strategy in periods of remission as well. This might increase the risk of nutrient deficiencies and malnutrition. According to the guidelines of the European Society for Clinical Nutrition and Metabolism (ESPEN), there are no reasons to introduce elimination diets during periods of remission [5]. Patients without active IBD have the same nutritional needs as healthy individuals. Therefore, they should not eliminate any foods without clear indications. Additionally, exclusion diets cannot be recommended to achieve remission in active IBD, even if a patient suffers from individual intolerances [5]. This systematic inquiry revealed insufficient evidence to make recommendations for exclusion diets as induction therapy. Exclusion diets have been described to reduce symptoms [2]; however, only a few uncontrolled studies have reported induction of longer remission [37,38]. In a randomized, controlled trial, maintenance of remission was seen in patients who used balanced diets and excluded foods that worsened symptoms, as compared to patients who received corticosteroids while following a regular diet [39]. Similar results on maintenance of remission were reported in an open-label study involving the same group following a personal food-exclusion diet [40]. There are no findings to confirm that exclusion diets are hazardous when applied rationally and under supervision. No evidence was provided to indicate that they contribute to nutritional deficiencies. Nevertheless, it is necessary to control potential nutrient deficiencies that might arise from any dietary exclusions.

Many studies show that IBD patients often suffer from malnutrition and vitamin and mineral deficiencies during phases of clinical remission and relapse [18,20,23,41]. This is, among other reasons, caused by restricted elimination diets that are often self-prescribed by patients. In our study, 53.7% of patients were on special (mainly lactose-free) diets, although none of them had confirmed lactose intolerance. Almost 66% of the patients admitted that their diets may lead to nutrient deficiencies, and 45.1% of the patients took vitamin or mineral supplements, not consulting a doctor. According to the ESPEN guidelines, only IBD patients with active disease and those who receive steroids, serum calcium and 25(OH) vitamin D should be monitored and supplemented, if required, to help prevent low bone-mineral density [5].

The idea that patients with IBD should resign from intakes of certain foods is quite controversial. According to the International Organization for the Study of Inflammatory Bowel Disease (IOIBD) and ESPEN guidelines, there is no specific diet that should be recommended to those patients. For that reason, the IOIBD created guidelines concerning each food group. The IOIBD recommends a moderate to high intake of vegetables and fruits and decreasing consumption of red meat for ulcerative colitis. On the other hand, the IOIBD suggests reducing saturated-fatty-acid intake. The IOIBD advocated against consumption of milk and unpasteurized products; however, no clear recommendations have been established with regard to consumption of pasteurized dairy products [17]. In the present study, fatty and spicy foods were mentioned as those most often eliminated by patients, along with dairy products, although only 28.9% declared being on lactose-free diets. Our patients declared withdrawals from some products not only during relapses of the disease but also during its remission. This may lead to unnecessary undernutrition. Similar results were found in other studies. Larussa et al. showed that 84% of patients with IBD avoided dairy products [29]. In a study by Marsh et al., participants most frequently reported avoiding spicy foods (46%), lactose-containing foods (41%), deep-fried/fatty foods (39%) and alcohol (38%) during active disease and spicy foods (39%) and lactose-containing foods (39%) during remission [27]. However, in a study by Vidarsdottir et al., the most frequently restricted foods were dairy products (60%), processed meats (55%), soft drinks (46%), alcohol (45%) and fast food (44%) [38]. Similar findings were reported by Casanova et al. The food groups avoided to prevent flares and during flares were spicy foods, alcohol, carbonated beverages and fiber [18]. In the study by Crooks et al., the most commonly eliminated foods were spicy and fatty products, carbonated drinks, milk products, alcohol, coffee and red meat [32].

During relapse, it is very common among IBD patients to restrict certain products [35,36,37,38]. The presence of gastrointestinal symptoms, the desire to reduce them, patients’ beliefs and experience were the most often-reported as possible reasons for avoiding some products [25,34,42,43]. In our study, some of the most often-eliminated foods were dairy products, which is consistent with other findings [35,37,38]. However, in clinical studies, no impact of reduction in dairy intake on IBD activity was observed [25,44]. One possible explanation why dairy products are eliminated from diets is spreading of information, both among patients and via the Internet, about the higher frequency of lactose intolerance in IBD patients. The fact that some IBD patients, most commonly CD patients, may develop lactose intolerance contributes to the fact that this nutritional restriction is often recommended. At this point, it is worth mentioning that a higher incidence rate of lactose intolerance among IBD patients is still controversial.

Although dietary counseling has been proven to be effective in reducing nutrition deficiencies in patients with IBD [18,39,45], only 30.5% of our respondents received any advice from a specialist (dietician, general practitioner, gastroenterologist, nurse). The majority of patients seek information about IBD diets on their own, mostly on the Internet, in books and in professional magazines. Among those who never received any information about IBD diets, 91.2% were willing to have dietary consultations. On the contrary, in the study by Zallot et al., 72.5% of patients received dietary recommendations, mainly from dietitians and gastroenterologists. In the study by Limdi et al., 49% of the patients received dietary advice [33]. Similar data were obtained in the study by Murtagh et al., in which 59% participants confirmed that they had received dietary recommendations. The majority reported that the source of this information was a dietitian. Each third participant was willing to be provided with further dietary advice [35].

The results of our study indicate that the majority of patients claimed that they had changed their diets after diagnosis; however, only 14.6% of the subjects believed that diet is more important in disease control than pharmacotherapy is. Other studies also verified patients’ behaviors after diagnosis. Their results were often divergent, and the reason for these differences remains unclear. A study by Maconi et al. reported that 38.6% of patients with CD and UC changed their dietary habits after diagnosis. The main dietary modifications were reduction in fatty and high-fiber foods and milk and cheese intake [46]. In the study by Limdi et al., 56% of patients modified their diets after IBD diagnosis and one-third believed that diet is more important in disease control than medications are [33]. In their study, Casanova et al. demonstrated that following IBD diagnosis, 66% of patients modified their dietary habits [18], whereas, in the study by Murtagh et al., 81% of patients were reported to have modified their diets upon IBD diagnosis [35].

We observed decreases in appetite and pleasure in eating during IBD relapses. Similar observations were made in other studies. Zallot et al. reported a reduction in the pleasure of eating in 47.5% of patients with IBD [34]. Patients who participated in the study by Limdi et al. felt that IBD affected their appetites (87% in CD and 66% in UC) [33]. The biological cause of reduced appetite in IBD seems to depend on many factors, such as disease phenotype, neuroendocrine influences and their impact on epithelial immune response, cellular homeostasis and nutritional impact on the gut–brain axis [47].

Food intake and specific dietary behaviors exert impacts on the everyday lives of patients with IBD [48,49,50,51]. Abdominal pain, functional gastrointestinal symptoms and food avoidance force patients to limit their social arrangements, which affects psychological health. In the present study, we did not observe diminished social life in our patients, in contrast to the findings obtained in other studies. The majority of patients included in our study shared meals with their families and did not avoid eating outside (bars, restaurants). In the study by Limdi et al., 25% of patients did not share meals with family members and 22% refused outdoor eating [33]. In the study by Zallot et al., 32.7% did not share meals with family members and 21.7% of patients refused outdoor dining [34]. However, in the study by Crooks et al., 59% of patients avoided sharing the same menu as the rest of their family and 58% refused eating in bars and restaurants due to fear of developing symptoms [32].

Our study had some limitations. All of the participants were recruited from a single center; most of them received biological treatment, which could have had an impact on their behaviors and beliefs. Therefore, these study outcomes do not directly reflect the overall IBD population in Poland.

## 5. Conclusions

Based on personal beliefs and experience, the majority of patients avoid certain foods during both relapses and remission of the disease, although such a management strategy is not supported by current scientific knowledge. Not only does this have a strong impact on their daily lives and psychosocial well-being, but it may also lead to nutrient deficiencies. Considering the growing interest in the role of diet in IBD treatment among patients, it is important that they are provided with firm dietary recommendations in order to avoid self-prescribed food restrictions. In regards to IBD control, patient education and disease knowledge should be the key determinants.

## Figures and Tables

**Table 1:** Characteristics of the study participants.

Characteristics	N (%)/Mean ± SD
Gender	
Female	42 (51.2)
Male	40 (48.8)
Mean Age (±SD) [years]	38.1 ± 11.6
Age Range	
<20 Years	3 (3.7)
20–40 Years	43 (52.4)
40–60 Years	32 (39.0)
>60 Years	4 (4.9)
Level of Education	
Primary	11 (13.4)
Secondary	31 (37.8)
High	40 (48.8)
Disease Type	
Crohn’s Disease	48 (58.5)
Ulcerative Colitis	34 (41.5)
Duration of Disease	8.4 ± 5.7
Smoking Cigarettes	
Never	46 (56.1)
Former	22 (26.8)
Current	14 (17.1)
Medications	
Biological therapy	66 (80.5)
Immunosuppression	33 (40.2)
Steroids	25 (30.5)
5-ASA	64 (78.0)
Disease Activity	
Ulcerative colitis	
Partial Mayo Score [0/1/2/3]	0 (0)/17 (50.0)/11 (32.4)/6 (17.6)
Montreal Classification	
Disease Location [E1/E2/E3]	4 (11.8)/16 (47.0)/14 (41.2)
Severity of Relapse [S0/S1/S2/S3]	12 (35.3)/10 (29.4)/11 (32.4)/3 (8.8)
Crohn’s Disease	
Crohn’s Disease Activity Index (CDAI) [0/1/2/3]	17 (35.4)/10 (20.9)/17 (35.4)/4 (8.3)
Montreal Classification	
Age at Diagnosis [A1/A2/A3]	8 (16.7)/36 (75)/4 (8.3)
Disease Location [L1/L2/L3]	17 (35.4)/7 (14.6)/24 (50)/0 (0)
Disease Behavior [B1/B2/B3]	21 (43.8)/18 (37.5)/15 (3.2)

**Table 2:** Patients’ beliefs.

Questions	Yes No. (%)
Do you believe that diet can initiate IBD?	27 (32.9)
Do you believe that diet can be a trigger for IBD flare?	70 (85.4)
Do you believe that diet is more important in IBD than medicines?	12 (14.6)
Do you think that your diet may cause nutritional deficiencies?	54 (65.9)
Do you think that you should avoid some products to prevent disease relapse?	67 (81.7)
Which products, in your opinion, should be avoided?	
- too spicy	44 (65.7)
- too fatty	33 (49.3)
- raw fruits	24 (35.8)
- raw vegetables	19 (28.4)
- green leafy vegetables	9 (13.4)
- leguminous	40 (59.7)
- cruciferous	32 (47.8)
- dairy products and milk	32(47.8)
- alcohol	32(47.8)
- citrus	9 (13.4)
- coffee	17 (25.4)
- vinegar products	10 (14.9)
- sweets	10 (14.9)

**Table 3:** Patients’ behaviors.

Questions	Yes No. (%)/Mean ± SD
Are you on special diet (vegetarian, lactose-free, gluten-free, low Fermentable Oligosaccharides, Disaccharides, Monosaccharides And Polyols Diet)?	44 (53.7)
- lactose-free diet	15 (28.9)
- lactose-free diet, but with cheese and yogurts	25 (65.8)
- gluten-free diet	2 (5.3)
Do you take any vitamins or minerals supplements?	37 (45.1)
Do you take probiotics?	15 (18.3)
Do you take any alternative therapies or herbal medications for IBD?	2 (2.4)
Have you modified you diet since the diagnosis of IBD?	62 (75.6)
Do you avoid some products to prevent the IBD relapse?	
- always	35 (52.2)
- often	20 (29.9)
- sometimes	12 (17.9)
- never	15 (18.3)
Does the IBD affect your appetite and pleasure in eating?	62 (75.6)
How do you evaluate your appetite during disease remission on a scale on 0 to 10?	8.7 ± 1.7
How do you evaluate your appetite during disease relapse on a scale on 0 to 10?	3.3 ± 2.6
During the relapse of IBD, do certain foods worsen your symptoms?	62 (75.6)
During the relapse of IBD, do certain foods reduce your symptoms?	12 (14.6)
Do you share the same menu with your family?	76 (92.7)
Do you refuse eating outside (restaurant, bar) for fear of causing relapse?	20 (24.4)
Have you ever skip the whole meal during the IBD relapse?	54 (65.9)
Have you ever skip the whole meal during the IBD remission?	9 (11.0)
Have you ever received any advice on your diet?	25 (30.5)
- general practitioner, gastroenterologist	10 (40)
- dietitian	14 (56)
- other IBD patients	1 (4)
How do you evaluate this advice on a scale on 0 to 5?	3.6 ± 1.2
Are you interested in receiving further nutritional advice?	52 (91.2)
Have you ever seek on your own for the information about diet in IBD?	55 (67.1)
- internet	51 (92.7)
- books, professional articles	4 (7.3)
How do you evaluate this information on a scale on 0 to 5?	3.3 ± 0.9

**Table 4:** Food exclusions among the study participants.

Food Groups	Yes No. (%)
Do you avoid some products during the IBD relapse?	74 (90.2)
- too spicy	50 (67.6)
- too fatty	49 (66.2)
- milk and/or dairy products	47 (63.5)
- gluten products	2 (2.7)
- raw fruits	26 (35.1)
- raw vegetables	25 (33.8)
- high-fiber products (leguminous, wholegrain products)	41 (55.4)
- alcohol	34 (45.9)
- coffee	25 (33.8)
Do you avoid some products during the IBD remission?	72 (87.8)
- too spicy	41 (56.9)
- too fatty	38 (52.8)
- milk and/or dairy products	39 (54.2)
- gluten products	3 (2.8)
- raw fruits	15 (20.8)
- raw vegetables	15 (20.8)
- high-fiber products (leguminous, wholegrain products)	31 (43.1)
- alcohol	30 (41.7)
- coffee	15 (20.8)

## Data Availability

Not applicable.
